# Adapting the “Faith Moves Mountains” Approach to Increase Dementia Treatment in a Rural Cohort

**DOI:** 10.1093/geroni/igaf122.1600

**Published:** 2025-12-31

**Authors:** Lisa Wiese, James Galvin, Jennifer Lingler, Ishan Williams, Janet Holt, Nancy Schoenberg

**Affiliations:** Florida Atlantic University, Boca Raton, Florida, United States; University of Miami, Coral Gables, Florida, United States; University of Pittsburgh, Pittsburgh, Pennsylvania, United States; University of Virginia, Charlottesville, Virginia, United States; Florida Atlantic University, Boca Raton, Florida, United States; University of Kentucky, Lexington, Kentucky, United States

## Abstract

Rurality confers a heightened risk for Alzheimer’s disease and related dementias (ADRD). Recent demographic shifts have fueled rural, small-town growth, reshaping the composition of rural communities with increased representation of older and racially and ethnically diverse individuals, whose ADRD risk is compounded by other factors. Using a community-based participatory approach, we tested the hypotheses that (1) a faith-based, resident-led approach would increase ADRD knowledge and screening, and (2) older age, female gender, lower educational levels, and more years lived rural would predict number of referrals, new dementia diagnoses, and treatment. An adaptation of Schoenberg’s (2009) Faith Moves Mountains model, previously successful in detection and management of other chronic illnesses in rural settings, guided this community-based participatory research. Local faith community members were trained as research assistants to recruit, administer surveys, conduct brief memory assessments, teach brain health strategies, and follow-up with residents. Outreaches were offered virtually during the pandemic, then in-person monthly at rotating church sites, and repeated ∼1 year later. This rural sample was 74.5% non-White, with 28% reporting

